# “Struggle at night – He doesn’t let me sleep sometimes”: a qualitative analysis of sleeping habits and routines of Hispanic toddlers at risk for obesity

**DOI:** 10.1186/s12887-022-03434-8

**Published:** 2022-07-13

**Authors:** Megan J. Gray, Christian E. Vazquez, Ojasvie Agnihotri

**Affiliations:** 1 Departments of Pediatrics and Department of Population Health, Dell Medical School, The University of Texas at Austin, 1601 Trinity St., Bldg B, Austin, TX 78712 USA; 2 School of Social Work, The University of Texas at Arlington, 211 South Cooper Street, Box 19129, Arlington, TX 76019 USA; 3 UT Health San Antonio Joe R. & Teresa Lozano Long School of Medicine, 7703 Floyd Curl Drive, San Antonio, TX 78229 USA

**Keywords:** Childhood obesity, Sleep, Hispanic, Primary care, Parenting, Night feeding, Infant and toddler nutrition

## Abstract

**Background:**

Hispanic children face disproportionately higher risks for early life obesity and resultant comorbidities such as Type 2 diabetes and fatty liver disease. Sleep habits are modifiable behaviors that impact early childhood obesity; Hispanic infants have been shown to have less nighttime sleep compared to their white counterparts. Pediatricians often coach families on parents’ nighttime responsive feeding and longer child sleep duration as protective factors against early life obesity, but must understand the family context and potential barriers. This study aimed to discover the sleeping habits and routines of Hispanic toddlers at risk for obesity through the perspective of their mothers.

**Methods:**

This qualitative study used a phenomenological approach. 14 Hispanic mothers were recruited from a Federally Qualified Health Center in Central Texas for qualitative interviews regarding their experience raising a small child. Children aged 6 to 18 months with child weight-for-length ratio ≥ 85% for age were approached for study involvement and consented during well child visits. Interviews occurred over several months during 2018–2019; NVivo software was used for analysis of qualitative themes. Two reviewers coded and used constant comparative methods to identify common themes.

**Results:**

Mothers diverged from AAP recommended guidelines for infant and toddler feeding and sleep habits. Mothers shared their intentions and the real-life barriers to implementing recommended habits. Mothers discussed wanting to have their child sleep in a separate bed or room but not having the resources (i.e., financial, space) to do so. Additionally, mothers discussed knowing not to feed to soothe at night but couldn’t bring themselves to let their child cry if they knew feeding would soothe them. Co-sleeping, feeding to sleep, middle of the night feeding, and lack of structured sleep habits were common interview themes and potentially modifiable factors.

**Conclusions:**

Pediatricians need to be sensitive to culture and the real-world needs of families to determine if best practices are “practical.” Themes from these parent interviews can inform tailored interventions for children at high risk of obesity. Interventions should promote responsive nighttime feeding and structured sleep, working with individual family logistics, to coach families towards optimal healthy environments and healthy child weight.

**Supplementary Information:**

The online version contains supplementary material available at 10.1186/s12887-022-03434-8.

## Introduction

Early childhood overweight and obesity is a growing public health problem, and an equity issue: it affects nearly half of all Hispanic children [1]. Additionally, Hispanic children face a disproportionately high morbidity for obesity-related negative health outcomes such as Type 2 diabetes or fatty liver disease, even in early childhood [2, 3]. An increasing body of evidence suggests that obesity, once entrenched as a diagnosis, is difficult to reverse; thus, preventative measures are most effective within the first “1,000 days” (prenatal to age two) [4]. Within this window, feeding and sleep habits are potentially modifiable behaviors that may be protective against rapid weight gain [5–7]. Pediatrician visits offer opportunities for counseling and parent coaching, but must be sensitive to the many systemic factors that influence families beyond the clinic. A qualitative exploration of Hispanic mothers’ experiences managing these behaviors may provide insights for pediatricians who work with Hispanic families.

### Role of sleep

Sleep medicine experts recommend 12–16 h of total daily sleep (including daytime naps) from four to twelve months of age; and 11–14 h from ages one to two years [8]. In addition to poor outcomes for emotional regulation and development, poor infant and toddler sleep duration has been associated with childhood obesity [7, 9–11]. Children who sleep less than ten hours at night are in the top quartile for obesity risk; possibly through a mechanism of greater energy intake associated with decreased sleep [5, 6]. Touchette showed that children sleeping less than 10 h per night have a four-fold risk of obesity by age six [12]; Taveras showed that infants sleeping less than 12 h per night is associated with two-fold risk of overweight at age three[13]. Later bedtimes are also associated with obesity, but odds may be attenuated by total sleep [14, 15]. There are cultural factors related to timing of daily routines: Hispanic children have delayed bedtimes when compared to their Caucasian counterparts as well as a shorter sleep duration [16]. In a recent study, Hispanic infants at six months slept 38 min less than their non-Hispanic white counterparts and were less likely to meet minimum sleep recommendations [17]. There is a need for research on the markers of sleep quality that may be associated with obesity and into the socioenvironmental factors that may preclude recommended sleep duration.

Sleep researchers make specific recommendations for infant sleep duration starting at four months; as prior to that age there is significant variability [8]; frequent parent responsiveness is also key to strong attachments [18]. From six to twelve months, there are fewer variations in sleep latency and duration; however as early as six months, 20–30% of infants experience sleep difficulties with frequent night wakings [19]. Nighttime wakings are influenced by parent behaviors: responding to night waking with feeding is most directly associated with infants’ not consolidating sleep [12]. Parents’ sleep-related behaviors such as presence when the child falls asleep, feeding to sleep, pacifier use, and room sharing, regardless of breastfeeding status, are associated with parent-reported infant sleep problems [20, 21]. Additionally, if parents feed at each nighttime waking, toddlers may increase their overall daily energy intake: predominantly nighttime feeding increased BMI-for-age Z score in 12–24 month children [22]. Number of nighttime wakings, or fragmented sleep, may also be associated with obesity risk through increasing serum cortisol [23].

Co-sleeping, defined here as bedsharing rather than room-sharing, has also been strongly associated with fragmented sleep for infants and toddlers; bedsharing is a strong predictor of nocturnal awakenings [12, 24]. Bedsharing is a parent behavior that may also increase the feeding-to-sleep association [20, 24]. The type of food offered at waking is also important: breastfeeding offered at waking is less obesogenic than formula [25], and co-sleeping is more closely associated with breastfeeding than formula feeding [26]. Co-sleeping varies by culture and was found to occur at a higher rate in Hispanic families compared to white families [27]. There are mixed studies on the association of co-sleeping and obesity; in a Danish study of 2–6 year old children, co-sleeping was associated with a decreased risk of overweight [28].

Pediatricians often make recommendations to parents that align with the American Academy of Pediatrics (AAP); however, what the AAP recommends isn’t always practical advice for some families. For example, AAP recommends against co-sleeping and promotes a studied set of specific safe sleep and nighttime behaviors for infants such as back sleeping, separate sleeping surface, and avoiding bedsharing, but promoting room-sharing until one year of age [29]. Additionally, the AAP does not specifically endorse sleep training (or “cry it out” methods), though they do encourage independent nighttime sleep by recommending phasing out nighttime feeding after six months of age [30]. These strategies may conflict with parent values and advice from that of other family members [31]. Parents rely heavily on their family members’ experience and cultural ties to develop their particular “parenting style” [32]. Notably, controlling and permissive parenting styles tend to be found in Hispanic families, and these parenting styles have been associated with overfeeding of young children [33–35]. Research into infant obesity prevention focuses on responsive feeding, recommending parents pay close attention to hunger and satiety cues, but limiting feeding at other times, i.e. an authoritative style [36]. However, research does not include attention to these behaviors (i.e., responsive feeding) at their intersection with sleep. For example, it is suggested that trained feeding-to-sleep has roots in overly responsive (permissive) parenting and this is an often understudied but important area of research that is relevant to the current study [37, 38].

### Cultural disparities in sleep and obesity

The socioecological model assumes that there are reciprocal interactions between individuals and their environment: an individual’s behavior is connected to the features of their institutions, community, and policy environment as well as interpersonal and intrapersonal characteristics [39]. Disparities in sleep and obesity in different racial and ethnic groups can be described using this framework. At the societal level, Americans value individualism and view a child sleeping in a crib or another room as “independence-training” [40], the majority of commercial sleep books in the United States support this view [41]. Additionally, emphasis on individual self-regulation is consistent with societal recommendations from large professional organizations, such as the AAP recommendations on following a bedtime routine, practicing self-soothing, and placing the infant in their own bed to sleep [42]. Alternatively, a number of parenting sleep books espouse bedsharing as integral to the breastfeeding dyadic relationship and strong attachment [26, 41]. The AAP also recommends room sharing until 12 months of age, which is at the height of infant separation fears and is past the window to optimize sudden infant death prevention, i.e. prior to six months; this contradicts the advice of sleep medicine experts to move the child earlier at six months [21]. Immigrant communities and other minority groups from collectivist cultures may value interdependence (and thus co-sleeping) more highly [43]. Such information can be contradicting, and parents turn to their friends and family for advice.

Beliefs regarding sleep vary amongst different ethnic communities in America, and Hispanic families have been found to have later bedtimes, co-sleep more frequently, and experience greater sleep curtailment [16, 27, 44]. Often times, families engage in parenting behaviors similar to the practices of those around them [43]. At the nuclear family level, Hispanic families tend to have lower socioeconomic status (SES) than that of non-Hispanic white families; financial stress may impact practical concerns regarding sleep [45]. For example, material hardship may result in longer and later work hours, variable shift work and decreased routine, and greater likelihood of bedroom-sharing due to a smaller home. Longer parent work hours (both mother and father) have been associated with increased childhood obesity [46, 47]. Bedtimes may also be impacted by SES and socio-cultural context. If parents arrive home from work in late evening, children may stay awake for a later mealtime or other nighttime family activities. All levels entrenched in the socioecological model influence these individual parent behaviors: leaving parents conflicted in their concern over pediatrician recommendations versus desires to follow cultural norms. Screening for such practices could identify opportunities for early obesity-prevention interventions.

Consideration of the sociocultural context and socioeconomic reality is crucial to understanding the constraints of each family and delivering recommendations that are feasible for them. There is a gap in the literature examining parents’ feelings about nighttime behaviors such as co-sleeping, nighttime routines, and nighttime feeding, particularly in a sample of low-income Hispanic mothers with children at risk for childhood obesity. Pediatricians offering well-intentioned advice need to be sensitive to the real-world needs of families, including their space and schedule constraints, to determine if best practices are “practical”, and how thus to best coach families towards optimal healthy environments.

### Rationale for this Study

Qualitative literature examining mothers’ experiences managing the sleeping habits and routines of Hispanic toddlers already at increased weight-for-length status is sparse. In 2015, Martinez and Thompson-Lastad conducted a similar study. focused on a sample of Latina mothers from California [48]. However, this study did not delve into infant feeding-to-sleep associations and motivations behind parent presence and middle of the night feeding. The current study aims to add to this study by possibly confirming their findings on nighttime routines and expanding on overnight feeding motivations a similar sample of mothers in Texas. Other studies have examined the intergenerational transmission of obesity in Hispanic families through overfeeding pathways without exploring in detail the motivations of parents with nighttime sleep and feeding habits [49]. Quantitatively, Ochoa and Berge’s literature review of home environment factors that contribute to childhood obesity, noted that deeper investigation into poor sleep and obesity in children is needed [50]. Thus, even though quantitative research has explored this area, more qualitative research on this topic is needed to get a deeper understanding of barriers for parents managing sleep behaviors of young children at risk for obesity. The current study comes from a larger study where we conducted qualitative semi-structured interviews to explore the parenting experience of Hispanic caregivers of infants and toddlers who were already at risk for overweight or obesity. In a previous manuscript using the same sample of 14 mothers, we found that parents describe ambivalence around recommended healthy behaviors, often struggle to provide structure while responding to the needs of their child, and meet with conflicting advice from partners and family; permissive parenting behaviors were common [51]. Sleep was a dominant theme, encompassing nighttime feeding, sleep location, and logistics of the bedtime routine. We hypothesized that parenting behaviors associated with obesogenic feeding may also extend to sleep behaviors, and aimed to better characterize the behaviors of our group. We also aimed to identify family behaviors around the sleep environment, schedule, and nighttime rituals that may be targets for intervention.

## Methods

### Recruitment and eligibility

Fifty families were initially recruited for a pilot study in a federally qualified health center, observing growth patterns of Hispanic children at risk for obesity. Of the 50 families, 15 consented to additional qualitative interviews. Parents were eligible to participate in interviews regarding parenting practices and experiences if they had a child aged 6 to 18 months with a weight‐for‐length ratio greater than or equal to the 85th percentile per WHO reference tables within the past 3 months, spoke English or Spanish, and if the parent/caregiver age was over 18 years. Exclusion criteria was prematurity (< 37 weeks gestation) or metabolic or genetic diagnosis affecting growth. Infants less than six months of age were not included because sleep patterns are not yet well established, and many infants are physiologically unable to go more than five hours without nighttime feeds before six months. Caregivers were recruited at routine well child preventive visits at a Federally Qualified Health Center in Central Texas which primarily serves Hispanic patients with Medicaid coverage. Informed consent was obtained and grocery store gift cards were given for study participation. Patients enrolled were both direct patients of the primary investigator as well as other patients of the practice. The research team included a pediatrician fluent in Spanish and bilingual Hispanic and English-only student interviewers. Following interviews, one family was later excluded for non-Hispanic ethnicity.

### Study procedures

At the point of recruitment, a demographic questionnaire was given to the caregiver in their language of preference, English or Spanish. The questionnaire that was developed specifically for this study contained demographic characteristics, and included 14 additional items on parenting behavior: 9 on feeding, sleep, and screen time behaviors; and 5 on program use (clinic and nutrition benefit programs). Interviews were conducted in the caregiver's language of preference in their home or another quiet location outside the clinic. Two graduate research assistants (1 male doctoral student and 1 female medical student) with training in qualitative methods conducted the interviews. The two interviewers were present at each interview to ensure important topics were probed if missed by one interviewer. We used a semi-structured 22-question interview protocol that was developed with input from a pediatrician and from other qualitative researchers in the first author’s department. Questions were intended to elicit mothers’ overall experience raising a small child. This phenomenological approach focused on key health indicators such as family support and neighborhood, feeding, play, sleep, and experience with the clinic. The section of the larger interview that focused on sleeping behaviors was introduced with the following question, “Tell me about what bedtime looks like for your child?” After three initial interviews, there were emerging themes such as feeding to soothe and justification of weight. To further explore these themes, we added nine questions to the interview protocol for a total of 31 questions. The broad sleep question above was followed up with the section specific general questions shown in Table 1. The interviews lasted between 30–60 min. We conducted interviews until thematic saturation was reached, which is appropriate for exploratory qualitative research. We chose to focus on sleeping themes for this manuscript, and a separate analysis of feeding themes has been published [51].

**Table 1:** Interview questions about infant sleep and routine practices

**General questions**
• How did you go about building your routine?
• Can you tell me about a typical night of sleep?
• Where does he/she sleep?
• How much time does he sleep at night or during the day?
• What time do you try to put him/her to sleep at night?
• Does he/she wake up crying?
• How do you lull him/her to sleep?
• When do you breastfeed?
**Probing questions**
• Can you tell me a little more about that?
• Will that change?
• For how long will they sleep with you?
• And how many hours?
• Have you tried other ways to lull them to sleep?
• And how much?

This study was reviewed by an ethics committee and approved by the University of Texas Institutional Review Board; participants signed consent forms before participating and were given assurance of confidentiality. Anonymity was not possible due to the nature of the research teams’ in-person interactions with the participants during recruitment and data collection; however, confidentiality was ensured by keeping audio recordings and transcriptions in encrypted password protected laptops. Identifying data was kept separate by using pseudonyms or participant IDs on documents and reports. The research team practiced reflexivity by writing memos and engaging in debriefing in order to attempt to set aside preconceived notions so that the analytic, substantive themes could emerge [52]. Participants were reassured that their participation in this qualitative study was voluntary and did not affect any other services or programs they were receiving at the clinic.

### Data analysis

The interviews were audio recorded, transcribed in the original language, and translated with verification from two bilingual researchers. The transcripts were uploaded to NVivo 12 for analysis. Two researchers read through the transcripts independently, following Creswell's analysis process, consisting of a first read‐through of transcripts to identify initial codes, then comparing initial codes condensing to final themes [52]. The researchers met periodically to discuss emerging themes, illustrative quotes, and areas of particular interest that appeared repeatedly. Based on the guidance from the literature, we determined that thematic saturation had been reached once the coders had reached the ability to obtain new information from the available data, which occurred after 14 interviews [53]. Once saturation was reached, the final identified themes were confirmed by all three members of the research team. Researchers later conducted descriptive quantitative analyses of sleep attributes (i.e., bedtime, hours of sleep, location of sleep) and height and weight data. Statistics regarding co-sleeping were gathered from semi-structured interviews because this was not clearly defined in our questionnaire. For analysis, we defined co-sleeping as bedsharing during any part of the night. There were no inconsistencies in audio recordings and transcriptions; participants were not followed up for corrections and were not consulted during the analytic process.

## Results

See Table 2 for demographic characteristics of study participants. Seven infants less than 12 months of age and seven toddlers 12–18 months old were included in the sample.

**Table 2:** Participant demographics (all Hispanic mothers), *N* = 14

Variable	n/%/avg./median
Language of preference	
Spanish	*n*= 11
English	*n* = 3
Age of Mother	
Age 22–26	*n* = 5
Age 27–33	*n *= 4
Age 34–41	*n *= 5
Median Age (years)	30
Relationship to Child	
Biological Mother	*n* = 13
Foster Mother	*n* = 1
Age of Child	
6–12 months	*n* = 7
13–18 months	*n* = 7
Median Age (months)	11
Child Gender	
Female *n* = 5	
Male *n* = 11	
Child Weight-for-Length Percentile	
Median	95.8%
Number of Children at Home ^a^	
One Child	*n* = 6
Two to Three Children	*n* = 4
More than Three Children	*n* = 4
Median Number of Children	2

*Note*. ^a^ Some siblings lived in home country and not in the home in the U.S

### Co-sleeping

**Fig. 1:**
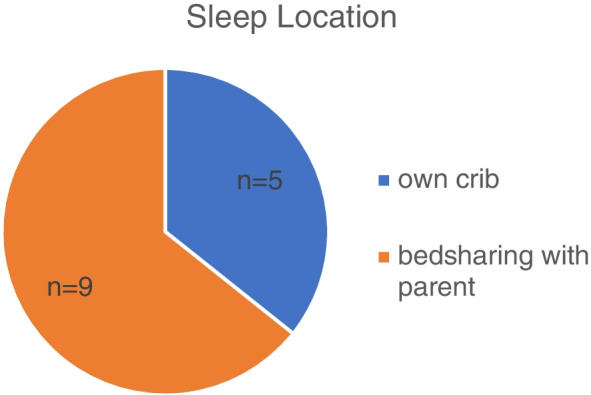
Frequency of regular sleep location; co-sleeping*, (*N* = 14). *co-sleeping defined as bedsharing with parent, not as room-sharing

The theme of co-sleeping included reports of mothers’ experience putting their child to sleep in the same bed where they sleep. In the questionnaire, 35% (*n* = 5) of caregivers reported co-sleeping (see Fig. 1), but 64% (*n* = 9) endorsed co-sleeping during their interview. About 60% (*n* = 8) of the mothers reported their child slept at least 9 h or more. See Table 2 for a description of the participants' demographic data and Table 3 for selected questionnaire items relevant to this study. Most commonly, mothers reported co-sleeping to lull their child to sleep and later moving them to their own crib. Some mothers reported having their child sleep in the crib at bedtime and later moving them into their bed as a reaction to their child’s arousal and difficulty falling asleep. Most mothers recognized that co-sleeping was not advised and intended to change this habit, but claimed that it was harder for their infant to fall asleep and stay asleep in their crib. Co-sleeping was often related to feeding at night since children often fell asleep with mothers after feeding. Both breastfeeding and formula feeding were equally associated with co-sleeping habits. Some mothers discussed not having the resources to have a separate crib and/or bedroom.

**Table 3:** Excerpt from questionnaire on household characteristics and sleeping habits

At what time does your child go to bed each night?7-8 pm // 8-9 pm // 9-10 pm // 10 pm +
How many hours of sleep does your child get on average**?** ^a^ < 6 // 6–7 // 7–8 // 8–9 // 9–10 // 10 +
Most nights, does your child sleep in their own bed, or with a parent? their own bed // with parent // other: __________________

*Note*. ^a^ Hours of sleep was not clearly defined here as nighttime but researcher clarified during interviews

### Nighttime feeding

The theme of nighttime feeding included reports of mothers’ experience feeding their child in the middle of the night after the child woke up crying. Our child participants’ median age was 11 months, and none of our interviewees were exclusively breastfeeding at the time of interview. About 70% (*n* = 10) of caregivers discussed waking in the middle of the night to feed their child. Feeding was often reported as a means of soothing their child to sleep when their child woke at night, indicating a feeding-to-sleep association. Mothers often reasoned that this behavior was an attempt to achieve adequate sleep for themselves, their partner, and/or their children. Some mothers recognized the need to stop nighttime feeding and expressed that they “want to take it away” from their child or that they plan to “work on that so it doesn’t happen.” Some mothers were also aware that their doctor advised against feeding during nighttime waking.

### Structured sleep habits

The theme of structured sleep habits included all nighttime and daytime rituals related to falling asleep, including sleep training, bedtime routine, and napping, as well as mothers who reported a lack of structured routine. While most mothers reported that their child fell asleep between 8 and 9PM on their demographic questionnaire, they revealed different responses during interviews, often mentioning their child falling asleep between 9 and 10PM. During interviews, 85% (*n* = 12) of mothers spoke about feeding their children at bedtime as a regular part of their bedtime routine. Mothers also reported many structured activities and rituals meant to relax their children at bedtime. Structured bedtime activities to encourage sleeping commonly included singing/music, turning off the lights, and feeding (solid food and/or milk). Some mothers reported difficulties establishing a daily sleep routine with timely naps that did not interfere with nighttime sleep. Mothers also reported ambivalence with implementing recommended sleep habits and routines and differences with partners; i.e. “he wants to give her some milk so that she will go back to sleep”; however another reported partner help “it’s more practical for him to soothe her.” Some mothers reported strong difficulty with crying, indicating a permissive parenting style:” I don't want him crying throughout the night wanting the bottle and not going back to sleep, so I just let him have it.” Mothers also indicated knowledge and desire to implement correct behaviors: “we have to work with her on that so that it doesn’t happen.” Table 4 shows the themes described above as well as quotes from participants providing evidence of their occurrence in the interviews.

**Table 4:** Themes and selected quotes

Themes	Quotes
Co-Sleeping: *Subthemes:* • *Availability of resources* • *Breastfeeding* • *Convenience*	**Study ID 18 (18 months):** “We’re bed sharing right now…I used to section her side of the bed off with a pillow. Like a little border pillow even up against the wall, because she would see if she would get up against the wall. And then I have a crib in *my* room for her, but I just—it needed a mattress. It was so I never got the mattress. And that was part of the reason why we just never—never did that.” **Study ID 13 (15 months):** “He sleeps in his crib. And that’s the struggle because when I have to take him out of the crib and breastfeed him and put him back. There are times that he does sleep with us” **Study ID 15 (18 months):** “He wouldn't go [to] bed. He would not go to sleep. And he would cry. So, we would get him out and lay him next to us…Not for that long! Not forever! Well I hope not. Maybe until he’s three. Three’s a good age.” **Study ID 4 (6 months):** “Just, it’s not like she sleeps in the crib because I don’t fit, but first she falls asleep with me in my bed, and then when she is already asleep, I get up and I put her in her crib, and then she stays there.”
Middle of the Night Feeding *Subthemes:* • *Breastfeeding* • *Response to waking/ soothing with feeding (vs. self-soothing)* • *Feeding-to-sleep association* • *Need for parent sleep*	**Study ID 13 (15 months):** “**I struggle at night** because he likes to breastfeed. He sleeps a little bit, but I want to take that away from him because **he doesn’t let me sleep sometimes**. I want to take it away because he doesn’t let me sleep. He falls asleep and within two hours he wakes up and I breastfeed him some more, so he falls back asleep. Like that, he wakes up like four times in the middle of the night.” **Study ID 3 (15 months):** “Sometimes I let her cry, but sometimes since my husband has to work, he wants to give her some milk so that she will go back to sleep. I tell him no, that he shouldn’t. She’ll get used to it. She will go back to sleep.” **Study ID 11 (9 months):** “I try not to give her the milk, but sometimes she just cries. So, then I have to give her some so that she goes to sleep, but she doesn’t drink much. Like she only drinks a little bit, and it’s because she’s like antsy. So, we have to work with her on that so it doesn’t happen.” **Study ID 15 (18 months):** “He'll wake up two hours into the night requesting his first bottle. He'll wake up around 3:00 and then around 4:00 or 5:00 wanting a bottle…I've been told several [times] by his doctor that he needs to get rid of the bottle, but I don't want him crying throughout the night wanting the bottle and not going back to sleep, so I just let him have it.”
Structured Sleep Habits *Subthemes:* • *Sleep Training* • *Bedtime Routine* • *Lack of structured Routine* • *Daytime sleep timing*	**Study ID 18 (18 months):** “She always gets a surge of energy right before she gets tired; she usually takes her socks off. Like she just… that's how I know she's getting sleepy. [laughs] The socks start coming off, and she's gonna run around for like a few minutes and then at that point we start saying good night to everybody and go upstairs brush our teeth…And then put on some relaxing music, and make sure that's going. And then I just nurse her.” **Study ID 21 (13 months):** “No, if he finishes the milk at night and still doesn't sleep, I don't give him more milk [inaudible] because I don't want him drinking more…because…I don't want him…the doctor said not to give him too much.” **Study ID 11 (9 months):** “Sometimes my husband helps me. And well it’s more practical for him to soothe her because well she sees me, and she wants to feed…So, he helps me, he gets up, he starts cradling her, singing to her, and talking to her until she falls back to sleep.” **Study ID 3 (15 months):** “Sometimes she also sleeps in the evening. I think that’s where I’m making a mistake, because people tell me that I shouldn’t let her sleep in the evening…That’s why she takes so long to sleep at night.”

## Discussion

Infant sleep is a potentially modifiable risk factor for obesity; parents are the greatest factor in promoting infant sleep consolidation and duration during the first two years of life. This study aimed to discover the sleeping habits and routines of Hispanic toddlers at risk for obesity through the perspective of their mothers. This study acknowledges that research into infant feeding often leaves out the sleeping behaviors that are closely intertwined with responsive feeding, and with trained feeding-to-sleep behavior [54]. This study adds sociocultural context to parents’ concerns about their infants’ and toddlers’ sleep habits, illustrating the ambivalence mothers particularly may feel when faced with recommendations that are difficult to implement in a real-world setting. For example, mothers in this study often diverged from AAP recommended guidelines for infant and toddler feeding and sleep habits in the areas of co-sleeping, middle of the night feeding, and lack of structured sleep habits. As it relates to co-sleeping, a common reason was not having the space or financial means to have the child sleep in a separate room or bed. For nighttime feeding, mothers often acknowledged their permissive parenting style to give in to feeding the child to soothe even when they understood it wasn’t the best strategy. Lastly, almost all mothers reported a lack of structure throughout the day which resulted in variation in when and where the child falls asleep at night. These insights present opportunities for pediatrician-family coaching.

Many mothers responded that they are co-sleeping (defined in this study as bedsharing): there is a difference in reactive co-sleeping due to necessity of limited sleep spaces, versus intentional co-sleeping to promote attachment [27, 29]. Pediatricians should ask *“Do you have a crib for her to sleep?”* More mothers acknowledged co-sleeping in interviews than in surveys; perhaps theyknew this went against pediatrician recommendations [20], or perhaps they responded as to their intent at the start of the night rather than the reality of responding to night waking. Well-intentioned pediatricians may ask unhelpful leading questions; for example: “He always sleeps in his own crib, right?”. A suggested script would normalize this difficulty and empathize with parents, i.e.: *“It can be hard to get babies to sleep comfortably in their own crib. Where does your baby sleep most often?*” If a child most often sleeps with parents; pediatricians can encourage parents to start with the child napping in their own crib, and then move to nighttime sleep. Pediatricians should be mindful to ask about unobserved sleeping in carseats, rockers, or swings, as these are not safe sleep approved but may be used as strategies by tired parents [29, 42]. Future studies would further assess how often children change sleep location during the course of the night, as well as clear differentiation of infant bedsharing versus room sharing with parents. We were not able to draw associations between the age of the mother or number of children and practicing healthy sleep habits.

There is also a need for further studies on sleep coaching in the Hispanic population; mothers interviewed did not seem to be working through a particular strategy to achieve sleep consolidation and limit nighttime waking and reflex feedings. Nighttime feedings – either used as the final step to soothe their baby to sleep initially or to soothe them upon waking at night—were identified as a particularly difficult habit to break. For this reason, infant sleep experts recommend teaching parents an “eat-play-sleep” routine so infants learn to separate feeding and sleeping [55, 56]. Targeting the feeding to sleep association, particularly in low income or non-native-English speaking families, is an unmet need in parent coaching for an obesogenic behavior.

The feeding to sleep association starts from an early age and is a [54]. It is key to maintain cultural sensitivity and acknowledge that changing parenting style or family habits is not intended as a rapprochement of culture. Simply asking about overnight feeds is a first step, “*How many times do you usually feed your baby overnight?”;*with special attention given to children over one year old who are still waking to feed at night. Parents can be counseled that if the child is growing well, this is habit waking as opposed to a genuine hunger. Parents need concrete tips to break the feeding-to-sleep cycle. For example, pediatricians can encourage parents to put babies down “drowsy-but-awake”; so that they learn to complete falling to sleep on their own and do not always rely on parent feeds when they wake at night [42]. This is an excellent opportunity to involve the other caregiver; particularly if there is a primary caregiver whom the infant associates more often with feeding. Also, parents can be taught to allow infants to practice self-soothing, one of the strategies recommended in the National Academies 2020 feeding guidelines on infants and children 0–24 months [36]. In practice, this means to wait a beat to see if the baby will settle before responding immediately; to react with a continuum of speaking softly, patting, or holding the child, before moving reflexively to soothing with feeding [24, 37, 42]. For infants who are healthy and growing well, a step further might recommend cutting down to one feed at night after six months of age, and no further nighttime feeding after nine months of age. To do this, parents can decrease the ounces or minutes of feeding slowly over a span of several nights. This stepdown approach is also more physiologic for maintaining a breastfeeding relationship, i.e. for the AAP-recommended 12 months and beyond [26, 57], compared to complete cessation of overnight feeds at four months that many commercial sleep training books recommend [18, 41]. Children still taking a bottle of cow’s milk several times a night after 12 months would be a red flag for intervention; as this is a risk for rapid weight gain as well as anemia [36, 58, 59]. Ensuring that babies have adequate “wake windows” and protecting daytime naps can also help ensure that a baby is not overtired, which can make nighttime sleeping without associations difficult [55, 56].

Pediatricians are trained to treat the patient in front of them in the exam room; only recently has there been a shift to “social medicine” in training and considering a patient’s full socioecological framework. In addition to considering the breastfeeding dyad, a closer investigation of partner roles, support and influence would also be helpful in exploring dual parent decision-making. Our study focused specifically on Hispanic mothers as they were the parent more often present at bedtime; however the role of fathers is now being studied with greater interest in affecting family functioning and infant self-regulation [60, 61]. For example, our parent who cited partner involvement at bedtime felt secure and supported in her planned bedtime structure; other studies show that another caregiver involved is beneficial to structured bedtimes, in addition to improved parent mental health [62]. We often ask about other caregivers during the day, but not specifically “*Who else helps you with feeding? Do you have help at nighttime?”*It can be helpful to ensure that different caregivers stick to a consistent routine to help with child habit forming. More information regarding household characteristics would be helpful in identifying room-sharing, the availability of other sleep spaces, the presence of electronics in the room, family schedules including parent shift work, and other modifying factors that could affect the infant’s sleep routine. Later bedtimes are common in Hispanic culture but are also associated with increased weight gain in young children [14, 16]. Multiple jobs, inflexible and irregular work hours can be a major barrier: parents with irregular work hours want to see their children when they return home late in the evening. These families could focus on morning family time rather than evenings (storytime or bath could be moved to the morning, and family breakfast instead of dinner); as well as quality over quantity, with undivided attention and special time with that parent on weekends or off days. More studies are needed on parent work schedules associated with family routines and early childhood sleep quality. There are likely specific factors for our Hispanic population, many of whom are also recent immigrants and dealing with financial insecurity, lack of family support, as well as acculturation logistical concerns, that may make the described sleep routines difficult to implement. Additionally, rising housing costs in Central Texas also makes roomsharing much more common for our sample. Attempting to change entire family routines and moving bedtimes may be the most challenging recommendation; family-friendly policies are needed to help with workplace accommodations for parents. A further area to explore also includes changes to routine with the COVID-19 pandemic with potential changes to work hours and caregiver availability.

### Strengths and limitations

There were several strengths and limitations of this study. Previous studies examine the importance of consistent bedtime routines and not bedsharing to improve infant and toddler sleep; other studies examine overall sleep quantity in the context of obesity prevention. This study specifically adds context on middle of the night feeding behavior in a population already at high risk of child obesity; examining parents’ motivations and barriers around feeding-to-soothe behaviors. This is consistent with Messayke’s 2022 study on parent behaviors that may affect infant sleep; however our study is novel to examine a Hispanic population within the United States, while examining the complex interplay of infant self-regulatory development and parent self-efficacy.

One area of research that we were not able to assess but perhaps plays an important role in the relationship between sleep behaviors and childhood obesity is an examination of nighttime formula intake compared to nighttime breastmilk intake; of the seven infants in our sample, only one was exclusively breastfeeding at the time of interview. Additionally, nighttime feeding was not specifically quantified in the questionnaire and was difficult to capture in interviews, with answers ranging from “every night” to “sometimes” to “not every night”. Actigraphy would be a more useful measures in future studies compared to self-reports. A limitation of the methods was that participants were not consulted during analysis. Including participant feedback during the analysis could have strengthened the credibility of the findings and would be closer to community-based participatory research. However, the research team followed a rigorous coding process to arrive at the final themes. Another limitation that may have influenced the findings is that we did not interview fathers. Since participants were recruited during child medical appointments, it was often the mothers that took the child to these appointments and thus no fathers were recruited from the eligible population. Obtaining fathers’ perspectives has proven to be a difficult task in this area of research but it critical to gain a deeper understanding of the child’s development. Many factors such as employment, other children in the home, single vs dual parent household, and more may affect structure throughout the day that ultimately affects sleep. Thus, conceptualizing structure in families’ life was a difficult theme to gain deeper insights because it varied so widely. The authors intend to further examine structure in a series of studies that is being carried out by the first author. A continuation of this study would obtain permission to contact families again about toddler and preschool sleep habits, any current sleep challenges, and to learn what has been most useful for families promoting healthy sleep behaviors.

## Conclusion

As sleep is a common topic causing concern for caregivers and affecting the well-being of the household, pediatricians should be comfortable coaching parents through sleep routine logistics and sleep training: using safe approaches that fit within the family’s goals and routines. Ideally pediatricians will work with families to develop reasonable solutions that move towards improved sleep habits, and soothing without reflex feeding: earlier bedtimes and structured sleep; decreasing overnight feeds after six months; all caregivers can be encouraged to take part in the child’s bedtime routine. This study also adds context around habitual nighttime feeding and obesogenic feeding-to-sleep behavior that disrupts infant self-regulation, and explores parenting motivations and barriers around changing this specific behavior, particularly for infants who are growing well and no longer in need of calories overnight. Particularly in populations at higher risk for obesity and obesity-related complications later in life, sleep should be a key part of a well child visit, its own “vital sign” for optimal infant growth and healthy development. Additionally, beyond the clinical setting, pediatricians can advocate for improved policies to support healthy family routines. Paid parental leave and paid time off, flexible work schedules or preferential scheduling for parents, and living wages would help with the sociostructural factors that currently undermine childhood sleep and health.

## Supplementary Information

**Additional file 1.** Raw data – interview transcripts publicly.

## Data Availability

Interview transcripts deposited in public repository: Harvard Dataverse.
