# Supplementary material for: “Struggle at night – He doesn’t let me sleep sometimes”: a qualitative analysis of sleeping habits and routines of Hispanic toddlers at risk for obesity
Source: BMC Pediatr. 2022 Jul 13;22:413. doi: 10.1186/s12887-022-03434-8 (PMC9277846; doi:10.1186/s12887-022-03434-8)
Supplement: Supplementary file 1 — Additional file 1. Raw data – interview transcripts publicly. [file 12887_2022_3434_MOESM1_ESM.docx]

**Supplementary files:**

Raw data – interview transcripts publicly available here:

Gray, Megan, 2022, “Qualitative interviews on sleep”, <https://doi.org/10.7910/DVN/IBNC2T>, Harvard Dataverse, v1
